# Fast synthesis of platinum nanopetals and nanospheres for highly-sensitive non-enzymatic detection of glucose and selective sensing of ions

**DOI:** 10.1038/srep15277

**Published:** 2015-10-30

**Authors:** Irene Taurino, Gabriella Sanzó, Franco Mazzei, Gabriele Favero, Giovanni De Micheli, Sandro Carrara

**Affiliations:** 1 Laboratory of Integrated Systems, EPFL - École Polytechnique Fédérale de Lausanne, Lausanne, Switzerland; 2 Department of Chemistry and Drug Technologies, Sapienza University of Rome, Italy

## Abstract

Novel methods to obtain Pt nanostructured electrodes have raised particular interest due to their high performance in electrochemistry. Several nanostructuration methods proposed in the literature use costly and bulky equipment or are time-consuming due to the numerous steps they involve. Here, Pt nanostructures were produced for the first time by one-step template-free electrodeposition on Pt bare electrodes. The change in size and shape of the nanostructures is proven to be dependent on the deposition parameters and on the ratio between sulphuric acid and chloride-complexes (i.e., hexachloroplatinate or tetrachloroplatinate). To further improve the electrochemical properties of electrodes, depositions of Pt nanostructures on previously synthesised Pt nanostructures are also performed. The electroactive surface areas exhibit a two order of magnitude improvement when Pt nanostructures with the smallest size are used. All the biosensors based on Pt nanostructures and immobilised glucose oxidase display higher sensitivity as compared to bare Pt electrodes. Pt nanostructures retained an excellent electrocatalytic activity towards the direct oxidation of glucose. Finally, the nanodeposits were proven to be an excellent solid contact for ion measurements, significantly improving the time-stability of the potential. The use of these new nanostructured coatings in electrochemical sensors opens new perspectives for multipanel monitoring of human metabolism.

## Introduction

Recent decades have seen an increase of the research on new high-performance devices, in particular for medical diagnosis, environmental control and pharmaceutical applications^1^,^2^,^3^,^4^. Electrochemical sensors offer considerable advantages as compared to conventional analysis methods (e.g., calorimetric, piezoelectric and optical), in light of their versatility, simplicity, low cost, capability of detecting compounds in real time, and the possibility of easy miniaturisation. The need to obtain new devices with high sensitivity, excellent selectivity and long stability over time has stimulated researchers to combine the advantages of electrochemical devices with those of nanomaterials^5^,^6^,^7^, which exhibit unique physical and chemical properties distinct from bulk materials. Very recently, platinum (Pt) nanostructures have been utilised to build electrochemical sensors because of their high surface area and their high electrocatalytic efficiency^8^,^9^,^10^,^11^,^12^. Their nanoscale size has been proven to enhance electrochemical sensing, particularly of kinetically-controlled electrochemical events^13^,^14^,^15^. For instance, the H_2_O_2_ electrooxidation occurring at Pt electrodes under a mixed diffusion and kinetic control, increases when the Pt is nanostructured^16^,^17^,^43^. It is worth noting that the majority of glucose electrochemical sensors are based on the indirect detection of H_2_O_2_, the product of specific enzymatic reactions. Park *et al.*^16^ demonstrated that Pt nanostructures increase electrode sensitivity to an extent that could allow enzymeless sensing of glucose, where electrooxidation is kinetically controlled. In addition, Pt-based nanostructured films exhibited near-Nernstian behaviour, fast response time and high precision when used as solid contact for ion-selective electrodes^18^. In the literature, different techniques to obtain nanoporous Pt have been reported so far, such as template and surfactant syntheses, and dealloying methods^19^. Ideally, an efficient approach to synthesise Pt nanostructures should be simple and free of surface contaminants but the above-mentioned synthesis procedures involve complicated preparation and costly materials^20^. Electrodeposition is the most efficient method to deposit nanostructures on electrodes due to its compatibility with electrochemical sensors and to the possibility of selectively confining nanomaterials onto electrodes. Among different electrodeposition protocols, the template-free method is without doubts the simplest, fastest and cheapest^2^,^19^. However, in template-free electrodeposition, the control of the nanoplatinum shape and size has not been studied in-depth.

In this work, we propose the very first method to prepare differently-shaped and well-ordered Pt nanostructures on a platinum substrate by a one-step template-free electrodeposition process using both four-and two-valent chloride-complexes hexachloroplatinate 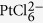 and tetrachloroplatinate 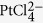
21. To further improve the electrochemical properties of the electrodes, depositions of Pt nanostructures on already nanostructured Pt electrodes was performed. All the modified electrodes were proven to be extremely powerful for a wide range or applications, ranging from potentiometric detection of ions to glucose sensing with and without an immobilised enzyme.

## Synthesis from tetravalent platinum

**Figure 1:**
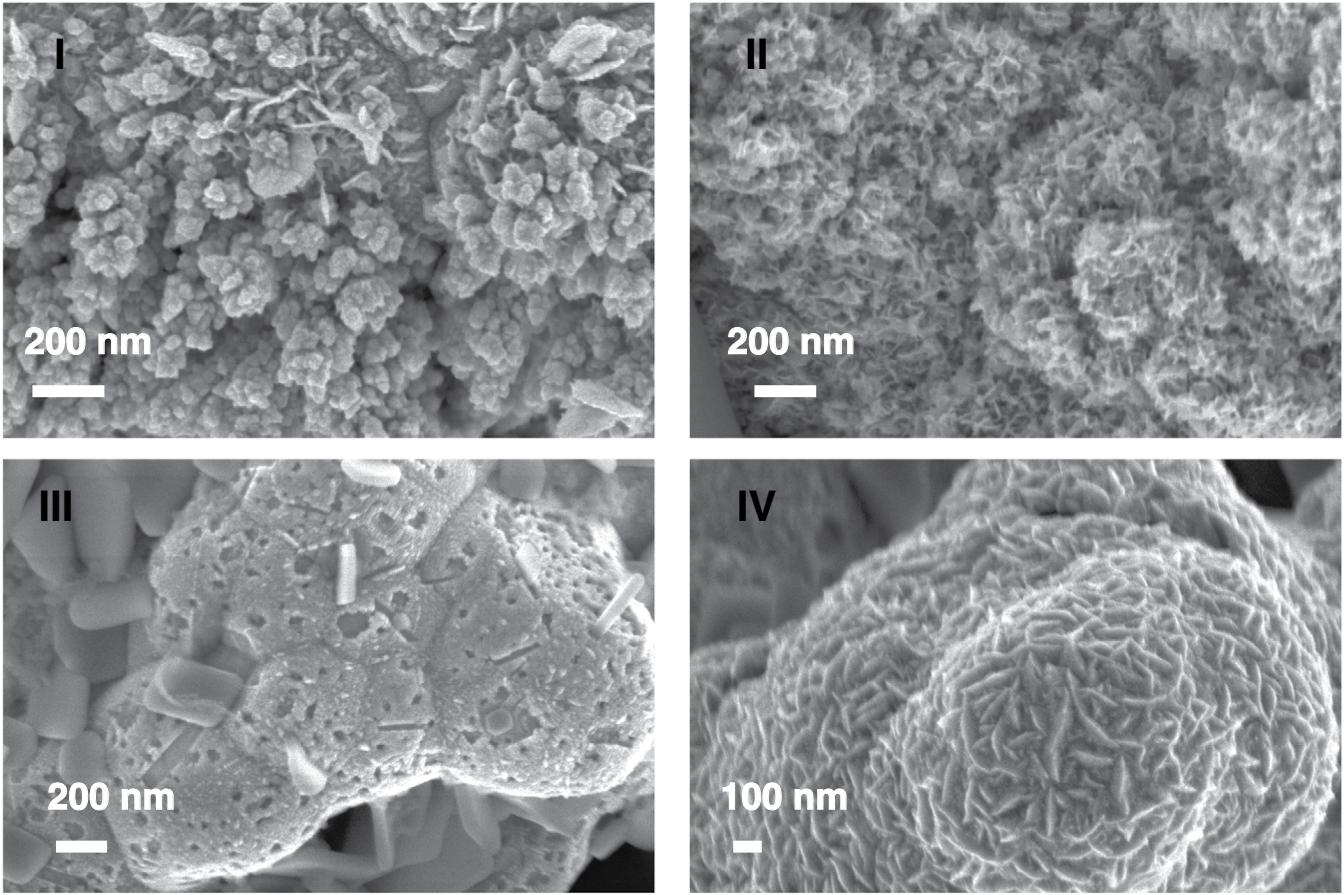
Morphology of electrodeposited Pt films after experiment I, II, III, IV.

**Figure 2:**
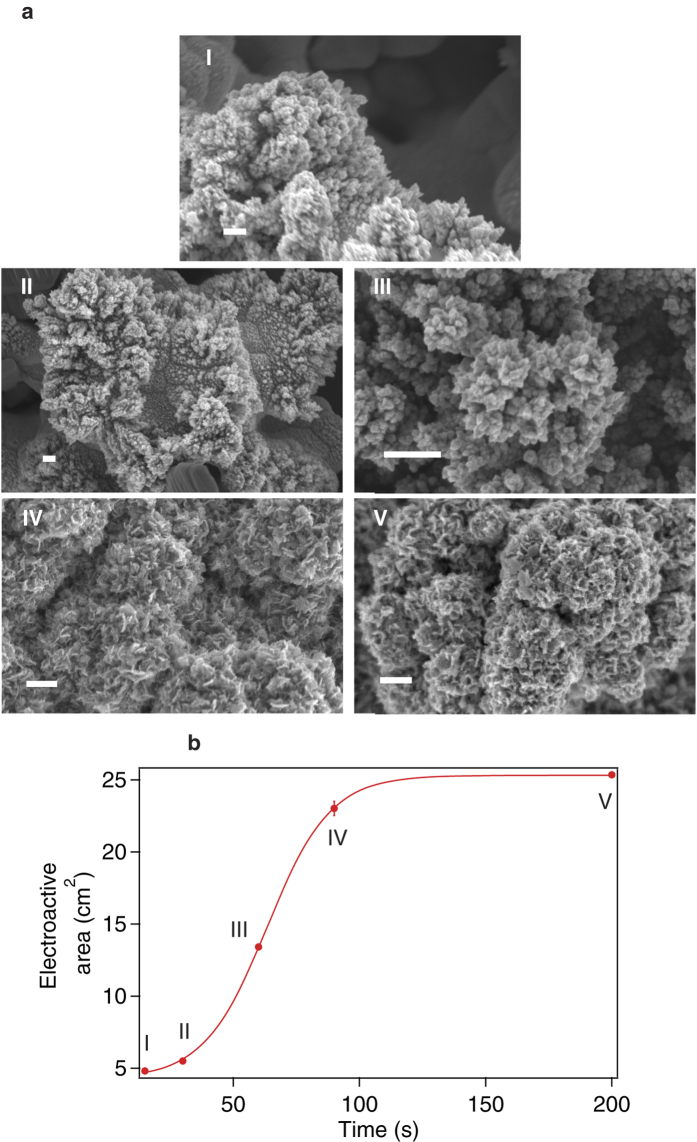
SEM images of Pt coatings obtained at different deposition times (bars: 200 nm) (**a**). Evolution of the electroactive area with deposition time (potential: −1 V; solution: 25 mM H_2_PtCl_6_ and 50 mM H_2_SO_4_) (**b**).

Pt nanopetals with average size of (68 ± 20) nm (Fig. 1 in Supplementary Information) were produced by applying −1 V for more than 90 s from a solution of 25 mM H_2_PtCl_6_ and 50 mM H_2_SO_4_. We obtained Pt flower-like nanostructures similar to those obtained by a chemical reduction with NaBH_4_^22^. Our nanostructures adhere strongly to the electrode and were advantageously obtained by a one-step deposition process. To study the influence of the deposition parameters on the formation of nanopetals, we used an experimental design called the Taguchi method. Three experimental parameters with two levels of variation were considered, leading to four experiments, according to a L4 Taguchi matrix^23^. The Taguchi technique allowed us to design and perform only a limited number of experiments, and to identify the principal factors influencing the results of the process^23^. The three deposition parameters were: applied potential (−1 V and −0.2 V), H_2_PtCl_6_ and H_2_SO_4_ concentration (3 mM and 500 mM, and 25 mM and 50 mM, respectively) and electrodeposition time (90 s and 200 s). The parameters used for each experiment (labeled I, II, III, IV), the respective electroactive area, density and dimension of petals can be found in Table 1 of the Supplementary Information. *Scanning Electron Microscope* (SEM) images in Fig. 1 show the morphology of the obtained nanostructures. From experiment I, Pt with a very low density of petals was obtained. An electrode fully covered with Pt nanopetals with the smallest dimensions (65 ± 16) nm was observed after experiment II was carried out. The electrode modified according to the parameters in experiment III does not display any nanostructure. Electrodes half-covered with big nanopetals ((142 ± 37) nm) were obtained in experiment IV. Such electrode has an electroactive area of (7.9 ± 0.1) cm^2^ that was 4-fold lower than the electrode obtained in experiment II ((25.4 ± 0.1) cm^2^). These findings confirm the fundamental role of both the petal density and size for a significant increase of the electroactive surface area. Then, we studied the influence of all synthesis parameters on the deposit characteristics (Fig. 2 in the Supplementary Information). The major factor influencing both petal density and electroactive area is the concentration of sulphuric acid and Pt salt in solution. Zhang *et al.* already observed the importance of H_2_SO_4_ in solution in Pt nanopetal synthesis^9^. In fact, the anions of the acid selectively adsorb on specific Pt surface planes, favouring their growth and resulting in an anisotropic material. In this work, we found that also the ratio H_2_PtCl_6_/H_2_SO_4_ plays a determining role on the Pt nanopetal growth. The higher the ratio is, the likelier is the nanosynthesis to occur. Depositions from a solution with the highest H_2_PtCl_6_/H_2_SO_4_ ratio produce a film covered by Pt nanopetals having the largest electroactive area (Experiment II and IV). The applied potential seems to play the most important role in defining the petal size. It is well-know that, at high overvoltages, depletion zones form around Pt particles, so that the distance between secondary nuclei increases resulting in the growth of ramified structures^19^. We demonstrated that the higher the absolute value of the potential is, the smaller the nanopetal dimension is. The effect of time on the electroformation of Pt nanopetals was also investigated. We carried out a set of syntheses at −1 V from a solution containing 25 mM H_2_PtCl_6_ and 50 mM H_2_SO_4_ (the conditions resulting in the highest density of nanostructures with the smallest size). It is clearly evident from Fig. 2(a) that spherical structures are obtained by 30 s of electrodeposition. High density, sharp-cornered nanostructures start appearing after 60 s of applied voltage^9^, corresponding to an active area halfway between the one of an electrode modified by 15 s of electrodeposition, and that of an electrode fully-covered by Pt nanopetals. The active area *vs* deposition time graph (Fig. 2(b)) displays a sigmoidal trend. Interestingly, by using the electrode almost totally covered with nanopetals, and obtained by applying −1 V for 90 s, the electroactive area was 85% of the maximum value. The maximum value of active area is predicted to be obtained after (167.5 ± 2.5) s of electrodeposition, corresponding to the time at which nanopetals cover the whole electrode surface. This value was extrapolated from a sigmoidal fitting.

## Synthesis from divalent platinum

**Figure 3:**
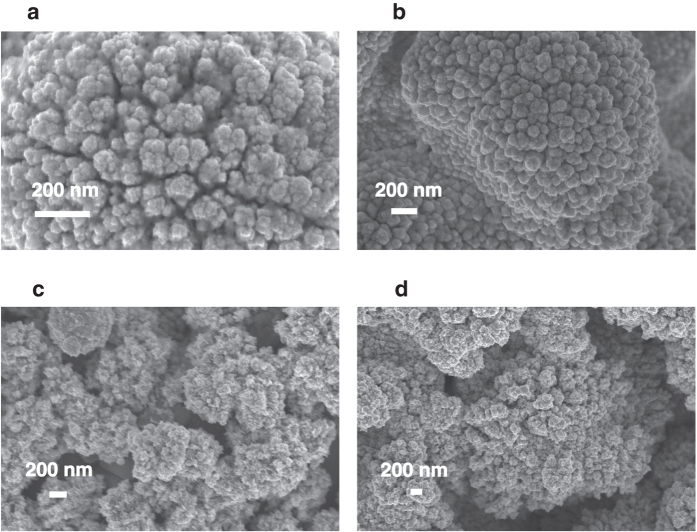
SEM images of Pt electrodeposited by applying −0.2 V for 90 s (**a**) or 200 s (**b**), and −1 V for 90 s (**c**) or 200 s (**d**), from solutions containing 25 mM K_2_PtCl_4_ and 50 mM H_2_SO_4_ (**e**).

The effect of applied potential and deposition time on the formation of nanostructured films was investigated by using a solution containing a divalent platinum salt (25 mM K_2_PtCl_4_ and 50 mM H_2_SO_4_). The morphology of the obtained deposits are shown in Fig. 3(a–d). Regular nanospheres with the lowest diameter are obtained by applying the lowest voltage (−0.2 V) for the maximum time (200 s). The average diameter of the structures evaluated from SEM images is (52 ± 18) nm. A similar value was obtained from AFM images ((51 ± 16) nm - see Fig. 3 in the Supplementary Information). Interestingly, these structures (Fig. 3(b)) have the highest active area among the electrodes (Fig. 3(a,c,d)) modified from K_2_PtCl_4_-based solutions. The electroactive area of Pt nanostructures in Fig. 3(b) ((24.9 ± 0.5) cm^2^) was similar to the the best value obtained from the petal-like nanostructures. This result confirms the importance of both size and amount of nanostructures for a larger electrochemical surface area. We also found that by increasing the deposition potential up to −2 V, nanopetals grow even from K_2_PtCl_4_-based solutions. The anions present in the electrodeposition solution inhibit more the reduction of divalent Pt than tetravalent Pt to Pt(0). Indeed, divalent Pt requires the availability of several adjacent sites because of its planar geometry, whereas tetravalent Pt is inhibited to a lesser extent because of its 3D geometry and its requirement for a single available site^24^. This phenomenon explains why Pt nanopetals form at a lower potential when using H_2_PtCl_6_, rather than K_2_PtCl_4_, at equal concentrations. We also observed that an increase of divalent Pt concentration from 25 mM to 37.5 mM K_2_PtCl_4_ in solution, leads to bigger nanospheres (from (47 ± 1) nm to (70 ± 1) nm - see Fig. 4 in the Supplementary Information), due to the availability of more Pt ions for reduction^25^.

## Subsequent depositions

**Figure 4:**
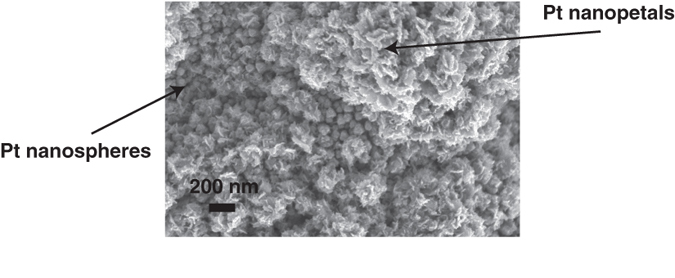
SEM images of Pt nanoflowers electrodeposited on nanospheres.

For further improvement of the electroactive area of the electrodes, we carried out two successive depositions. After the deposition of nanospheres (−0.2 V, 200 s, 25 mM K_2_PtCl_4_ + 50 mM H_2_SO_4_) we synthesised Pt nanopetals onto the overgrown electrode (−1 V, 90 s, 25 mM H_2_PtCl_6_ + 50 mM H_2_SO_4_). Pt nanopetals grown on nanospheres are shown in Fig. 4. The presence of the two differently shaped nanostructures is clearly evident. The double-coated electrode has an excellent active area ((28.2 ± 0.4) cm^2^), even higher than the largest area obtained with one-step depositions of both nanospheres and nanopetals.

## Non-enzymatic oxidation of glucose

**Figure 5:**
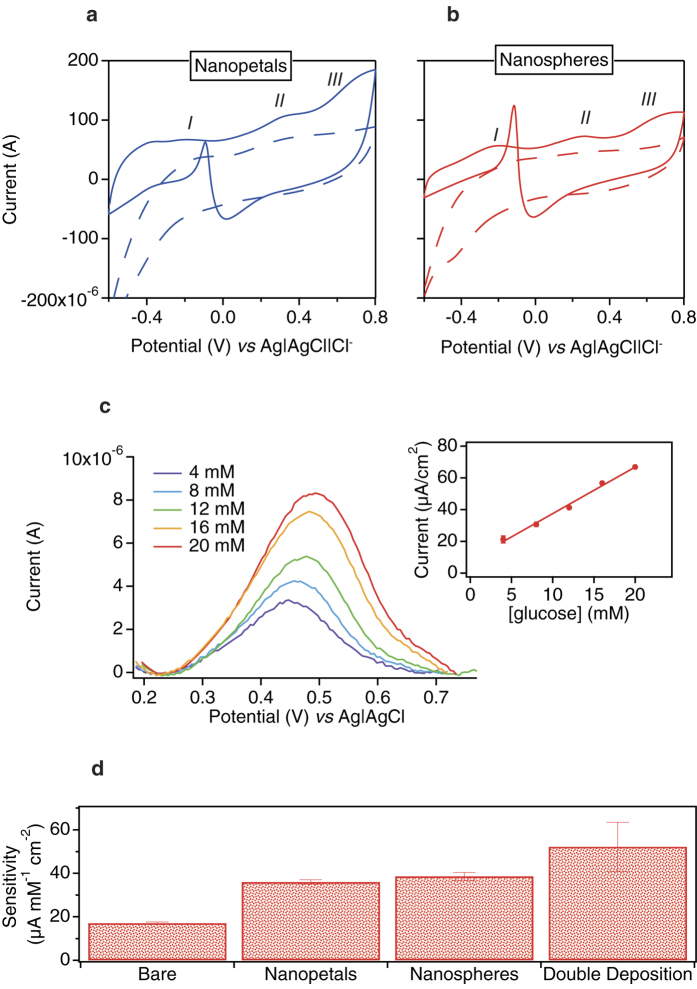
CVs at 20 mV/s of electrodes modified with nanopetals ((**a**) in blue; −1 V; 200 s, tetravalent Pt-based solution) and with nanospheres ((**b**) in red; −0.2 V; 200 s, divalent Pt-based solution) in solutions containing PBS without (dotted line) and with (straight line) 20 mM glucose. *Square wave voltammetries* (SWVs) at 15 mV/s in cell media containing glucose concentrations equal to 4, 8, 12, 16, 20 mM and respective calibration curve (estimation of standard deviation from duplicate measurements) (**c**). Sensitivities to glucose detection of enzyme-based electrodes without and with nanostructures, namely, Pt nanospheres (−0.2 V; 200 s, Pt-based solution), Pt nanopetals (−1 V; 200 s, tetravalent Pt-based solution), and Pt nanopetals (−1 V; 90 s, tetravalent Pt-based solution) grown on Pt nanospheres (−0.2 V; 200 s, divalent Pt-based solution). Error bars refer to the standard error of inter-sample triplicate measurements (**d**).

We investigated the capability of the nanostructured electrodes to detect glucose without an immobilised enzyme. The three glucose oxidation peaks, clearly evident in Fig. 5 for Pt nanopetals (a) and Pt nanospheres (b), demonstrate the capability of the modified electrodes to retain a high sensitivity towards the non-enzymatic detection of glucose. In fact, generally, bare platinum electrodes suffer from poisoning by adsorbed intermediates and from poor selectivity for glucose detection. It is worth noting that *cyclic voltammetries* (CVs) were acquired in the presence of physiological concentrations of chloride and phosphate anions, both well known inhibitors of glucose adsorption onto the Pt surface and, therefore, of relative oxidation^26^,^27^. The three typical glucose oxidation peaks were detected with the nanostructures were found to be similar to those reported in the literature^26^. It is worth mentioning that Pt nanostructures promote and enhance sluggish reactions like glucose oxidation^26^. The highest and most defined peaks were acquired with Pt nanospheres-based electrodes. This may be attributed to the presence of a greater number of active sites for the direct electrooxidation of glucose on Pt nanospheres-based electrodes. The importance of the nanocatalyst structure has previously been demonstrated for the enhancement of ethanol oxidation^28^. Therefore, Pt nanospheres were chosen for enzymeless measurements of glucose directly in cell media. Due to the poisoning effect, peak current II, commonly used to detect glucose at Pt electrodes^2^,^5^,^28^,^29^, is reduced when measuring glucose in media rather than in PBS. However, an increase of the peak current was acquired for increasing glucose concentrations within the glucose physio-pathological range. Sensitivity was (2.9 ± 0.1) μA/(mM cm^2^), a value higher or comparable to those obtained by other authors in synthetic buffer^2^,^5^,^28^,^29^ (Fig. 5(c)). To the best of our knowledge, this is the first-ever reported example of direct glucose detection in undiluted cell media.

## Oxidase-mediated detection of glucose

*Chronoamperometry* (CA) was employed to determine the electrochemical response of differently nanostrucured electrodes. In this case, we used an indirect detection of glucose *via* H_2_O_2_ electrooxidation (applied potential: +700 mV *vs* Ag|AgCl|Cl^−^). A glucose oxidase was immobilised by the crosslinking method onto the electrode and the oxidation current of a reaction product (H_2_O_2_) was monitored for a comparative study of the signal response. It was found that all the nanostructured electrodes exhibited a higher electrochemical response towards the indirect glucose detection relative to bare electrodes. The sensitivity of the Pt nanopetals and the Pt nanospheres was threefold that of bare electrode. A slight improvement was recorded by using the hybrid electrode with Pt nanopetals on Pt nanospheres (see Fig. 5(d)). These findings indicate that the nanoporosity of the modified electrodes favours H_2_O_2_ electrooxidation, that is half kinetically-controlled, resulting in a higher electrochemical signal for sensing glucose than unmodified electrodes. Table 1 shows the sensitivities of biosensors based on GOx and Pt nanostructures for glucose detection *via* H_2_O_2_ electrooxidation. Only Wang and coworkers have built first generation glucose biosensors based on H_2_O_2_ electrooxidation using nanoporous Pt^17^. Other authors have extensively reported the use of Pt nanoparticles mainly on carbon nanomaterials to improve the sensing performance of this class of biosensors. The sensitivity we obtained is higher than those reported in other works using Pt nanostructured electrodes.

## Solid-contact ion-selective electrodes

**Figure 6:**
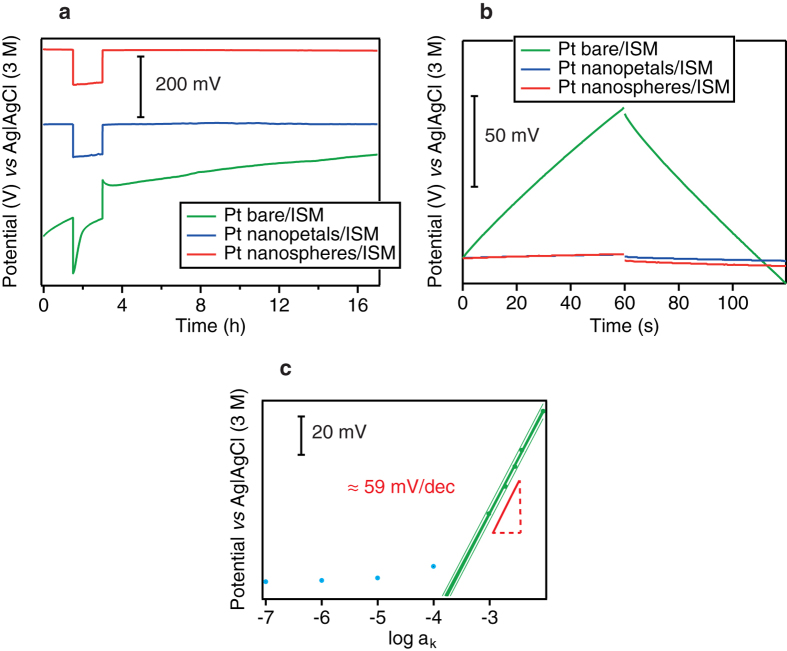
Water-layer test for Pt-K^+^/ISM electrode (green line), Pt nanospheres-K^+^-ISM electrode (red line; −0.2 V for 200 s from divalent Pt-based solution) and Pt nanopetals-K^+^-ISM electrode (blue line; −1 V; 200 s, tetravalent Pt-based solution) (**a**). RCP for Pt-K^+^/ISM electrode (green line), Pt nanospheres-K^+^-ISM electrode (red line) and Pt nanopetals-K^+^-ISM electrode (blue line). The applied current was +5 nA for 60 s and −5 nA for 60 s in 0.1 mM KCl (**b**). Calibration plot (in green) of the solid-contact K^+^-selective electrode based on Pt nanostructured electrode (**c**).

Nanostructured electrodes exhibited not only a higher electroactive area as compared to bare electrodes but also an important redox capacitance. This additional property makes these nanostructured films excellent candidate intermediate layers between an electrode and an ion-selective membrane (ISM) for the fabrication of solid-contact ion-selective electrodes (SC-ISEs) with enhanced time stability. We cast a PVC K^+^ ISM^30^ onto a bare electrode and onto an electrode modified with Pt nanopetals and Pt nanospheres. Potentiometric water tests were carried out to evaluate the formation of a water-layer between the membrane and the solid contact. Figure 6(a) shows the potentiometric response of the bare electrode and of the two nanostructured ones in 0.1 M KCl, 0.1 M NaCl and again 0.1 M KCl. Only with the nanostructured electrodes no drift was found when switching the solution back from Na^+^ to K^+^. They exhibited a better stability over time (Pt nanopetals: (32 ± 2) μV/h; Pt nanospheres: (98.8 ± 3) μV/h) relative to bare Pt ((7698 ± 5) μV/h), comparable with the best results reported in the literature^31^,^32^,^33^,^34^. The short-term potential stability and the electric capacitance of the electrode were also studied by *reversed chronopotentiometry* (RCP). Figure 6(b) shows a typical RCP for electrodes with and without nanostructuration. From the potential jump, we estimated the total resistance of the electrode according to Ohm’ s law (R = ΔE/I). The resistance decreased by one order of magnitude when nanostructures were included as solid contact, passing from ≈3 MΩ to ≈0.3 MΩ. The short-term potential drift, computed from the slope of the plot E *vs* t (ΔE/Δt), was (1441 ± 107) μV/s for electrodes without nanostructures and (33 ± 2) μV/s and (42 ± 7) μV/s when Pt nanopetal- and Pt nanosphere-based electrodes were employed, respectively. The low frequency capacitance was computed according to the equation ΔE/Δt = I/C and was estimated to be (152 ± 9) μF and (120 ± 19) μF for nanopetal and nanosphere-based electrodes, respectively. These values are two orders of magnitude higher than those related to bare electrodes ((3.3 ± 0.3) μF). Based on these findings, we can argue that we built a very stable solid-contact K^+^ ISE, based on electrodeposited Pt nanostructures. Electrodeposited nanoporous Pt has been previously used as solid contact to build pH^35^ and reference electrodes^18^. However, these authors employed a template method to build the nanoporous film. In our work, the nanostructured layer was obtained with a one-step procedure, simpler and faster than the method employed by Park^35^ and Han^18^. We also investigated differently shaped Pt nanostructures as solid contact for ion sensing. The calibration plot for K^+^ detection shown in Fig. 6(c) demonstrates that the ion-selective electrode based on our Pt nanostructures exhibits a near Nernstian behaviour.

In summary, we produced Pt nanostructures with high surface areas on bare Pt electrodes by a simple one-step template-free electrodeposition method. Using hexachloroplatinic and tetrachloroplatinic acid and changing both deposition potential and deposition time, we obtained spherical and petal-like nanostructures. Modified electrodes exhibit a higher performance than bare electrodes for enzyme-mediated glucose sensing. The three characteristic glucose oxidation peaks were well-defined only with the nanostructured electrodes. A markedly stabler potential was acquired with the modified electrodes than with bare Pt, when potentiometric ion measurements were carried out. The simpler and faster preparation procedure than those used by other authors^18^,^35^, combined with the promising sensing and biosensing results of the Pt nanostructured electrodes, suggests that the present modification approach could have a great potential for various electrochemical applications, especially in the advanced monitoring of metabolism where low-cost, highly integrated, and multipanel devices are highly desired, in the field of health diagnostics^1^,^36^ as well as cell biology^37^,^38^.

## Experimental Methods

### Electrodeposition procedures

Pt nanostructured electrodes were prepared using Autolab potentiostat under a computerised control (Metrohm, Switzerland) from solutions containing H_2_SO_4_ (95–98%, Sigma) and Pt salts, H_2_PtCl_6_ (Aldrich) or K_2_PtCl_4_ (FisherSci). Depositions were carried out by applying −0.2 V or −1 V at room temperature while stirring. A carbon electrode was used as counter electrode and placed in parallel to the working electrode. A silver (Ag) electrode was used as pseudoreference. Coatings were deposited on Pt *screen printed electrodes* (SPEs) (Metrohm; 12.56 mm^2^), or on Pt electrode microfabricated by metal evaporation (7.14 mm^2^), the latter used only for potentiometric measurements. All bare electrodes were cleaned before the nanostructuration step by applying +2 V for 60–120 s. After the deposition process, a material activation was carried out, consisting in acquiring multiple CVs between −0.2 V and +1.5 V at 100 mV/s in 0.1 M H_2_SO_4_ till the overlap of two subsequent voltammograms^18^. Then, the cleaning procedure was repeated.

### Morphological characterisation

The morphology of the obtained nanostructures was investigated with a Zeiss Merlin high resolution scanning microscopy and a Bruker Atomic Force Microscope. Petal sizes and nanosphere diameters were computed with an ImageJ software (http://rsbweb.nih.gov/ij/) from SEM images and with Nanoscope Analysis software from AFM images.

### Measurements

We computed the integral of the peak related to the Pt oxide reduction in CV acquired in 0.1 M H_2_SO_4_ (Autolab, Metrohm, Switzerland) using IgorPro software (Wavemetrics, Lake Oswego, OR, USA)^39^ after subtracting the electrochemical double layer current density. We computed the charge value (Q) by dividing the integral in A V by the scan rate (V/s). The value of the electroactive area of Pt nanodeposits was calculated as Q (mC)/0.42 mC where 0.42 mC is related to an atomically smooth Pt surface of 1 cm^2 ^39.

CVs for the direct glucose oxidation were acquired in a 0.01 M PBS solution (Sigma, pH 7.4) in a potential window between −0.6 V and +0.8 V at 20 mV/s. SWVs were acquired at 15 mV/s in cell media (Dulbecco’s Modified Eagle’s Medium - DMEM, D5030, Sigma). Chronoamperometric intersample measurements of glucose were carried out in triplicate at +700 mV *vs* Ag|AgCl|Cl^−^ in PBS solution (Sigma, 10 mM, pH 7.4) under stirring and aerobic condition by consecutively adding glucose (Sigma) up to 500 μM by 100 μM steps, with SPEs with and without nanostructuration. Glucose oxidase-based solution (10 μl) from Roche (15 mg/ml in PBS with glutaraldehyde 2.5%) was cast onto the electrodes and kept dry at 4 °C before use. Calibrations were carried out with IgorPro software. Inter-sample measurements were taken in triplicate and the standard error of the resulting sensitivities was computed.

K^+^ ISM was obtained by dissolving 33.5% high molecular weight poly(vinyl chloride) (PVC, Fluka), 65% bis(2-ethylhexyl)sebacate (DOS, Fluka), 0.5% potassium tetrakis(4-chlorophenyl)borate (KTClB, Fluka) and 2% potassium ionophore I (Fluka) in 1 mg tethrahydrofuran (THF, Fluka) per 100 mg of mixture. The membrane was kept dry for 24 h and then conditioned in 0.01 M KCl (Sigma) for 24 h before starting the experiments. All the potentiometric measurements were performed using a double-junction reference electrode (Metrohm, Switzerland, Ag|AgCl, 3 M KCl). Pt and nanostructured Pt were coated with a drop of K^+^ ISM (8 μl). The analytical performance of K^+^ selective electrodes was studied for concentrations ranging between 10^−7^ M and 10^−2^ M KCl. The activity coefficients were computed using the Debye-Hückel equation^30^.

## Additional Information

**How to cite this article**: Taurino, I. *et al.* Fast synthesis of platinum nanopetals and nanospheres for highly-sensitive non-enzymatic detection of glucose and selective sensing of ions. *Sci. Rep.* **5**, 15277; doi: 10.1038/srep15277 (2015).

## Supplementary Material

Supplementary Information

## Figures and Tables

**Table 1:** Performance of some biosensors based on GOx and Pt nanostructures for glucose detection *via* H_2_O_2_ electrooxidation.

Biosensors	Potential (V)	SensitivityμA/(mMcm^2^)	Ref.
GOx/Pt/CNT/TiO_2_	−	0.24	40
Pt/MWCNTs/GOx/GC	+0.60 *vs* Ag|AgCl	58.9	41
Nafion/Chit/GOx/Pt nanocubes/Pt	+0.60 *vs* Ag|AgCl	35.92	42
3D porous Pt nanowires/GOx	+0.55 *vs* Ag|AgCl	8.74	17
Pt nanopetals on Pt nanoflowers	+0.70 *vs* Ag|AgCl|Cl^−^	51.6 ± 10.8	This work
