# Supplementary material for: Fast synthesis of platinum nanopetals and nanospheres for highly-sensitive non-enzymatic detection of glucose and selective sensing of ions
Source: Sci Rep. 2015 Oct 30;5:15277. doi: 10.1038/srep15277 (PMC4626773; doi:10.1038/srep15277)
Supplement: Supplementary Information [file srep15277-s1.doc]

Fast synthesis of platinum nanopetals and nanospheres for highly-sensitive non-enzymatic detection of glucose and selective sensing of ions

Irene Taurinoa,∗∗, and Gabriella Sanzóa,b,∗∗, Franco Mazzeib, Gabriele Faverob, Giovanni De Michelia, Sandro Carraraa

aLaboratory of Integrated Systems, EPFL - École Polytechnique Fédérale de Lausanne, Lausanne, Switzerland

bDepartment of Chemistry and Drug, Sapienza University of Rome, Italy

Table 1 Taguchi experimental L4 array of deposition conditions (applied potential, composition of solution, electrodeposition time) and respective outputs (active area, percentage density and dimension of nanopetals).

∗Corresponding author. Irene Taurino Tel.: +41 21 69 30 917; Fax: +41 21 69 34 225 Email address: irene.taurino@epfl.ch

∗∗These authors equally contributed to the work.

Figure 1 SEM image of Pt nanopetals at low (a) and high (b) magnification (-1 V; 90 s; 25 mM H_2_PtCl_6_ and 50 mM H_2_SO_4_).

Figure 2 Response graphs of S/N ratio for larger-is-better analysis of electroactive area (a) and of nanopetal coverage area (b). Response graph of S/N ratio for smaller-is-better analysis of nanopetal dimension (c).

Figure 2 AFM 3D image of Pt nanopheres deposited at by applying -0.2 V for 200 s from divalent platinum solutions.

Figure 3 SEM images of Pt nanospheres deposited from solutions containing two different concentrations of K_2_PtCl_4_ and respective histograms of the sphere diameters.
